# *irreversibility*: A Python Package for Assessing and Manipulating the Time Irreversibility of Real-World Time Series

**DOI:** 10.3390/e27111146

**Published:** 2025-11-12

**Authors:** Massimiliano Zanin

**Affiliations:** Instituto de Física Interdisciplinar y Sistemas Complejos CSIC-UIB, Edifici Complex de Recerca de les Illes Balears, Parc Bit, E-07120 Palma de Mallorca, Spain; mzanin@ifisc.uib-csic.es

**Keywords:** time irreversibility, statistical test, time series, Python package

## Abstract

Time irreversibility refers to the property of some dynamical systems and time series of being statistically different when observed backward in time. While the theoretical foundations of irreversibility date back to the origin of statistical physics, the analysis of such property in real-world time series has only recently gained momentum. We present *irreversibility*, an open-source Python (version ≥ 3.11) package aimed at providing a large set of irreversibility metrics and tests and at facilitating their use. Besides the tests themselves, it includes a set of utilities, like functions to downsample and manipulate the time series, and to optimise the parameters of the metrics. By providing a unified software package, *irreversibility* simplifies the analysis of real data, allowing the researcher to compare multiple tests and obtain a better and more reproducible view of the underlying system. In this contribution we explore the features of the package and provide examples of its use.

## 1. Introduction

Time-reversal symmetry refers to the property of a system (or of a time series extracted therefrom) of being invariant, in a statistical sense, when the arrow of time is reversed, i.e., under the transformation t→−t. To illustrate, the dynamics of an ideal pendulum without friction is invariant under a time-reversal operation; by watching a movie, an observer could not tell whether the movie corresponds to the original (forward in time) dynamics, or to its time-reversed one. The same would happen in the case of a completely random dynamics, e.g., a time series corresponding to a white noise, as no structures associated with the arrow of time can be present; i.e., all values are independent from the preceding and following ones. It is nevertheless not difficult to find examples in which this symmetry is broken. This is the case, for instance, of the previously mentioned pendulum, but now with friction; observing the pendulum increasing its speed clearly indicates that we are watching the time-reversed dynamics. Other examples include a glass with ice cubes melting, as opposed to water solidifying to form regular ice cubes; or a flame consuming the fuel source.

The concept of irreversibility has a long history within statistical physics. In its beginning, it was a controversial topic: while it is undeniable that many processes we observe around us are irreversible, the fundamental physical laws (e.g., Newton’s laws) are strictly time-reversible. Such conflict between theory and observation was colourfully illustrated by M. Born in 1949: “*Irreversibility is a consequence of the explicit introduction of ignorance into the fundamental laws*” [1]. Since then, statistical physics and thermodynamics have helped elucidate how time irreversibility emerges—the interested reader can refer to ref. [2] for a historical account. Irreversibility is a characteristic feature of systems operating away from equilibrium, including living and active matter [3]. It has been shown that time reversibility implies stationarity [4], and that linear Gaussian random processes and static non-linear transformations of such processes are reversible. On the other hand, time irreversibility implies non-linear dynamics, and linear or non-linear non-Gaussian processes as possible generative mechanisms [4,5,6]. Memory breaks time symmetry, acting as a dissipative force. On the contrary, noise results in a loss of irreversibility [7]. Irreversibility is also strongly connected with causality [8], as one of the basic assumptions of the latter is that causes must precede effects—as leveraged by all predictive causality tests [9]. Hence, irreversibility has been associated with the arrow of time and causality in thermodynamics [10], and with the fluctuation theorem [11]. At the same time, it has been shown that the link between irreversibility and causality can be broken when studying deterministic (e.g., chaotic) systems [12,13].

In parallel to these theoretical advances, a more recent trend has been the development of metrics and statistical tests to numerically assess the irreversibility of a time series representing the dynamics of a real-world system. The objective is clear: by assessing the irreversibility of an observed dynamics, conclusions can be extracted about the underlying mechanisms generating it. For instance, if irreversibility stems from memory and computation, then brain activity should be irreversible, and such property may be modulated by different pathologies [14,15,16]. To this end, many tests have been developed—see for instance refs. [17,18] for reviews. In most cases, these tests are not only small variations on similar concepts. On the contrary, being the definition of irreversibility agnostic to the kind of statistical difference defining the arrow of time, these tests are based on highly different starting points. To illustrate, some of them detect asymmetry by applying time-asymmetric functions to the data [19]; by considering the order of values within small subwindows of the original series [20,21,22]; or even by mapping time series into complex networks [23,24]. Such heterogeneity supposes a challenge for any real-world data analysis: as different tests detect different patterns of irreversibility, many (if not all) of them should be applied in parallel—otherwise a system may be wrongly categorised as time-reversible and vice versa; see refs. [17,18] for examples. Many of these tests have free parameters that ought to be optimised to the problem at hand. Finally, software source codes have seldom been published, requiring an ad hoc implementation in each case. In short, the interested researcher will find the systematic evaluation of time irreversibility a challenging task.

In this contribution we present *irreversibility*, a freely-available and open-source Python package including a suite of 18 irreversibility tests for real-world data analyses. It further provides the researcher with tools to simplify the analysis, including the automatic optimisation of parameters; the downsampling of time series, thus supporting multi-scale evaluations; the manipulation of time series, to either increase or decrease their irreversibility; and the generation of synthetic time series using known models. Algorithms have been optimised to minimise the computational cost, by using both the Numba Just In Time (JIT) compiler [25], and GPU execution via shaders through WebGPU [26]. This package is thus intended to complement other solutions available within the Python ecosystem to study time series, including *TISEAN* for non-linear analyses [27], *pyunicorn* for network representations [28], *ordpy* for order patterns [29], or *tsfresh* for feature extraction [30].

In the remainder of the text we first describe the structure of the package and its main elements, including the full list of irreversibility tests and associated characteristics. Next we present a set of examples to show how such tests can used in real scenarios, exploring the functions offered by *irreversibility*. We conclude by analysing the computational cost, especially with respect to the two available optimisation options, and drawing some conclusions.

## 2. The *irreversibility* Package

**Figure 1:**
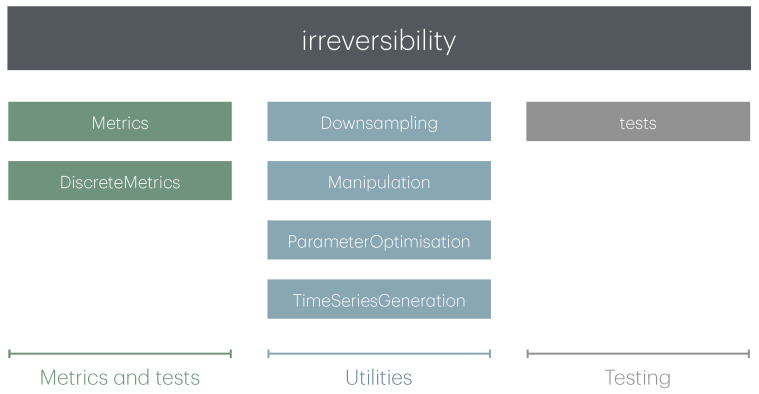
Schematic representation of the structure of the *irreversibility* package.

As represented in Figure 1, the global structure of the *irreversibility* package is organised around three pillars. Firstly, one finds its core: a collection of metrics and tests to assess the temporal irreversibility of time series, organised according to their nature (i.e., assuming time series composed of continuous or discrete values). Secondly, a set of utility functions to simplify the analysis, including tools for downsampling and for generating synthetic data. Thirdly, the package includes a set of Unit Tests, designed to validate the individual functions; this is not included in the main package distribution, but can be accessed by the interested user through the code repository. The first two pillars are expanded below.

### 2.1. Irreversibility Tests

For the sake of completeness, we report here a short description of the irreversibility tests included in the package. Note that additional information can be found in related reviews [17,18], and in the included references to the original papers. While a taxonomy of irreversibility tests is hitherto yet to be proposed, a general classification according to the type of analyses they perform is reported in Table 1. The majority of the tests are designed to analyse the irreversibility of real-valued time series, as this problem has received more attention in the literature—possibly for tackling a type of data that is more common in real-world applications. The full list (as for version 1.1.0) is reported below:

- BDS test [31]: Test proposed by Brock, Dechert, and Scheinkman for detecting low-dimensional chaos in time series, which has later been applied to time irreversibility. Note that a statistically significant result may indicate the presence of low-dimensional chaos, and not necessarily of a time asymmetrical dynamics. It is based on comparing two sample correlation integrals, respectively, at embedding dimensions *m* and 1, and assessing if the normalised difference follows a N(0,1) distribution.
- Casali [32]: It calculates a distance $d_{\left(i,m+i\right)}$ between points $x_{i}$ and $x_{m+i}$, i.e., between points that are *m* samples away from each other. The skewness of the distribution of *d* is then calculated; if this is statistically different from zero, the time series is labelled as irreversible.
- COP [22]: Test based on Continuous Ordinal Patterns, an extension of permutation patterns (see below) in which a distance is calculated between a given reference and sub-windows of the time series. Multiple patterns are tested, in each case evaluating whether the distance for the original and time-reversed time series are drawn from the same distribution.
- Costa Index [33]: Initially proposed to describe heartbeat dynamics, it is based on comparing the number of instances in which the time series increases or decreases. When dealing with real-valued time series, the amplitude of these changes is disregarded; a more complete version is also available for discrete values; see below.
- DFK [34]: Introduced by Daw, Finney, and Kennel, this test transforms the values of the time series into symbols; merges them together into “words”; and finally compares the probability of appearance of each word in the original and time-reversed time series.
- Diks [35]: Test based on evaluating whether two sets of vectors, extracted from the original and time-reversed time series, correspond to the same multi-dimensional probability distribution.
- Local Clustering Coefficient [24]: This test transforms the time series into a complex network [36,37] using the directed Horizontal Visibility Graph (dHVG) approach—as described below. The retarded and advanced local clustering coefficients are then compared, i.e., the propensity of the network to form triangles, respectively, backwards and forwards in time.
- MSTrends [38]: Test based on comparing the distribution of the slopes of linear fits (or the highest-degree coefficients in polynomial fits) obtained for small overlapping sub-windows of the original time series.
- Permutation Patterns [21]: Test based on the symbolisation of a time series using permutation patterns, i.e., the rank sequences corresponding to short sub-windows of the original series [39]. Irreversibility is then assessed by calculating the difference in the frequencies of each pattern and of its time-reversed version. Note that similar alternatives have independently been proposed, as, e.g., refs. [40,41,42].
- Pomeau [19]: One of the first tests for detecting irreversibility in time series; it is based on calculating a time-asymmetric polynomial function on the data, and on comparing the results between the original and the time-reversed version.
- Ramsey [43]: Test based on the comparison of the method-of-moments estimators of two sample bicovariances.
- Skewness [44]: Proposed by D. Koutsoyiannis, it considers the original time series and its differenced version; it then calculates an index of irreversibility as the relation between the skewness (i.e., the degree of asymmetry in the distribution) of the two versions.
- Ternary Coding [20]: Test based on symbolising the values of the time series, specifically considering three symbols (i.e., strongly increasing, strongly decreasing, and maintaining) for then evaluating the difference in their respective frequency under a time-reversal operation.
- TPLength [45]: Given the trend patterns of the time series, i.e., sequences of consecutive increasing or decreasing values, it calculates the irreversibility from the asymmetry in the probability distributions of their length.
- Visibility Graph [23]: This approach involves transforming the time series into a visibility graph, i.e., a network whose nodes represent the individual values, and links are created whenever the line connecting the values corresponding to two nodes is not obstructed by another intermediate point—yielding a directed Horizontal Visibility Graph (dHVG). Time irreversibility is then accepted if the distributions of the number of links arriving at and departing from nodes are different in a statistically significant way.
- Zumbach [46]: Test designed to detect irreversibility in financial time series; it firstly involves transforming the values into their returns; afterwards, two volatilities are calculated, i.e., a historical (in the past) and a realised (in the future) one.

On the other hand, the package includes the following tests for discrete-valued (i.e., symbolic) time series:

- Gaspard [47]: Test based on assuming that the sequence of symbols has been generated by a Markovian random process, such that two entropy rates can be calculated: a time-reversed entropy per unit time, and a standard (forward in time) one. The difference between the two yields an estimation of the entropy production, and hence of the irreversibility of the process.
- Costa Index, discrete version [33]: Full implementation of the index proposed in the original paper [33] and discussed above. This version assumes that the time series is composed of discrete values that can be ranked, i.e., that a difference can be calculated between them, as is for instance the case of time series of integer values.

An important point in the estimation of the irreversibility of real-world time series is the dichotomy between irreversibility measures and tests. All tests previously described yield both a statistic, quantifying the magnitude of the irreversibility, and a *p*-value, quantifying the corresponding statistical significance. Some tests considered here, for instance the BDS one, naturally return a *p*-value; others have trivially been adapted to support such tests; e.g., the Visibility Graph approach initially generates two distributions that can be compared using an Epps–Singleton test. A third category of measures is nevertheless more complex: those that only return a statistic, or a metric of the intensity of the time asymmetry, without presenting a natural way for extracting a *p*-value. *irreversibility* solves this by resorting to surrogate time series: once the metric is calculated, the same metric is also calculated over an ensemble of randomly shuffled versions of the same time series. Such shuffling destroys any temporal relation, and by definition, also any time irreversibility. The final *p*-value is then calculated in two ways:

- Proportional: Proportion (or fraction) of shuffled time series yielding a statistic equal to or higher than what is observed in the real time series. This approach presents the advantage of yielding a true *p*-value. Yet, it is also computationally very expensive if a high precision is required. To illustrate, using 100 random time series, the smallest *p*-value that can be obtained is 0.01.
- Z-Score: The value obtained with the real time series is compared to the distribution yielded by the randomly shuffled ones through a Z-Score, which is then transformed into a *p*-value. While computationally efficient, as good approximations can be obtained with as low as 20 random time series, the result is not a true *p*-value.

The availability of these three options (i.e., proportional, Z-Score, and native *p*-value) is reported for each metric in the last three columns of Table 2.

As a last point, some of the tests here included have a high computational cost, especially when applied to long time series—this topic will be further discussed below. While all measures are coded in standard Python, two additional options are provided: a Numba Just In Time (JIT) compiler [25] version, which involves pre-compiling the code and can usually yield between 10× and 100× speed-ups; and GPU execution via shaders using WebGPU [26]. The availability of these two options is reported in the third and fourth columns of Table 2.

### 2.2. Utilities

As previously introduced, the *irreversibility* package includes a set of utilities designed to support the user throughout the analyses, and to simplify some common steps.

These include, firstly, a set of methods for downsampling the time series, i.e., the common procedure to evaluate the multi-scale nature of the irreversibility—a comparison between them is available in ref. [18]. Three options are available: retaining one every τ observations; extracting sub-windows of size τ from the original time series, and substituting their values with the corresponding average; and a decimation process, based on downsampling the signal after applying a low-pass anti-aliasing filter.

Secondly, it offers an algorithm for the manipulation of the irreversibility of time series, i.e., modifying the data in order to increase or decrease its time asymmetry, while minimising the magnitude of the changes on the data themselves [48]. This is achieved by applying Continuous Ordinal Patterns [22] and by finding the one that manipulates the irreversibility in the desired direction, while maintaining a high linear correlation between the original and manipulated time series.

Thirdly, the package has a set of functions to optimise the parameters of each test previously presented. Note that this is relevant in real-world applications, as some tests are highly sensitive to these, and using wrong parameters can lead to an underestimation of the irreversibility. This parameter tuning is based on the assumption that the user wants to obtain the lowest possible *p*-value from a test; this is achieved by applying the test under study to a set of time series provided by the user, evaluating several parameter combinations, and selecting the one yielding the lowest *p*-value. Note that, in order to avoid overfitting, it is strongly suggested to divide the available data set in two parts; one of them is then used for tuning the parameters, and the other for evaluating the results. Additionally, the user must be aware that some specific applications may require different types of optimisations, such as finding the parameters yielding a maximum difference between two groups of time series.

Finally, *irreversibility* provides a large set of functions for the generation of time series of known irreversibility. These can be used both to test new time asymmetry metrics and to validate larger data pipelines. These include the asymmetric Weierstrass function [49]; the Henon, Logistic, and Linear Congruential Generator chaotic maps; the Lorenz chaotic system; the Ornstein–Uhlenbeck process; the geometric Brownian motion with stochastic resetting (srGBM) [50]; a model to generate time series that are irreversible at specific time scales, as proposed in ref. [18]; and a symbolic random walk.

## 3. Examples and Use Cases

We illustrate here how the package can be used to analyse the irreversibility of time series, focusing on some common use cases and applications. For the sake of clarity, these examples are organised below in subsections by goal.

### 3.1. General Irreversibility Analysis

We base the following examples on synthetic time series, both to illustrate how the corresponding functions are used and for the sake of reproducibility. As shown in Listing 1, after the initial importing of the corresponding library functions, both the generation of a synthetic time series using the logistic map and the assessment of its irreversibility using the COP approach take one line of code each.

**Listing 1.** Generation of a synthetic time series and calculation of its irreversibility through the
COP test.

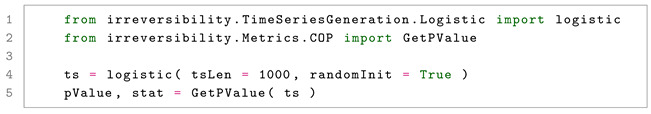



Note how the inputs and outputs of each test are formatted the same way, e.g., the output is always two floats with respect to the *p*-values and the statistic of the test. Consequently, changing the test only requires changing the corresponding import statement. To illustrate, the BDS test can be calculated using the code of Listing 2.

**Listing 2.** Calculation of the irreversibility through the BDS test.

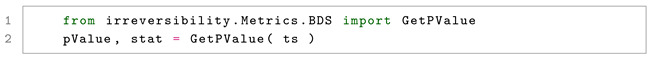



In the previous examples, the tests were executed using the corresponding standard parameters—note that no option is being passed, except for the time series under analysis. It is nevertheless important to take into account that tests may be highly sensitive to these parameters, up to underestimating (or even directly missing) irreversibility when wrong values are used. The package provides a method to perform an automatic optimisation, based on calculating the *p*-values on a set of time series using different parameters’ values, for then selecting the combination yielding the most statistically significant result.

In Listing 3, we first generate a list of synthetic time series, again using the logistic map here. Note that when analysing real-world time series, it is convenient to separate a subset of them for optimising parameters—i.e., akin to the split of data into training and testing common in machine learning validation [51]. When no additional instructions are provided, the function tests a set of possible parameter values (in the case of COP, size of patterns between 3 and 6 included) and yields the optimal one. For instance, in this case the output would be something similar to ({‘pSize’: 3}, np.float64(1.9777347755496739e-57)), where the first element is a dictionary with all the optimal values, and the second is the smallest achieved *p*-value. These parameters can then be passed to the irreversibility test, either manually or in an automatic way, as seen below in Listing 4:

**Listing 3.** Example of the optimisation of the parameter of the COP test.

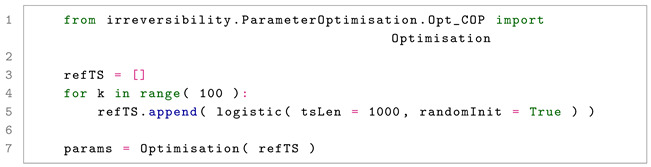



**Listing 4.** Passing parameters to the test functions.

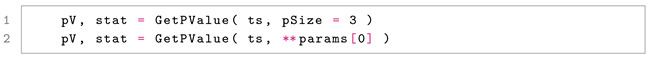



### 3.2. Automatise Across Different Tests

As highlighted in the literature, the existence of many time irreversibility tests responds to the imprecise definition of the same: it states that a difference is present between the original and time-reversed version of the time series, but provides no indication regarding its nature [17,18]. As a consequence, different tests may yield different results; and several of them have to be compared, in order to obtain a complete picture. Thanks to the fact that all tests use the same interface, it is easy to compare several of them. To illustrate, *irreversibility* provides a list, irreversibility.allTests, including pointers to all available functions.

Listing 5 exemplifies the process. In the first line it loads the list of functions; each element is composed of a string with the name of the test, and of a pointer to the corresponding test function. The code then iterates over them, and prints the result. The first five lines of the output are reported in Listing 6; note how all tests, except the DFK one, correctly identify the time series as irreversible. Regarding the latter, better results are obtained by optimising its parameters, as opposed to using the original values, as shown in Listing 3.

**Listing 5.** Applying multiple test functions.

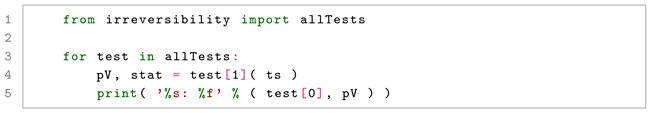



**Listing 6.** First five lines of the output of Listing 5.

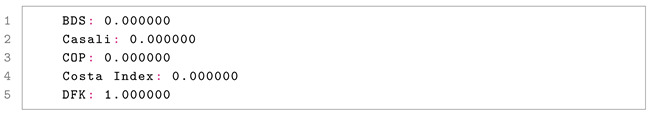



### 3.3. Time Series Downsampling

For the sake of completeness, the *irreversibility* package includes several functions to manipulate time series, specifically focused on multi-scale analyses. These usually involve downsampling the original time series, in order to evaluate the irreversibility across different temporal resolutions [18]. To illustrate this, Listing 7 imports the function Downsampling_Avg, which extracts sub-windows of size τ (here equal to two) from the original series, and substitutes their values with the corresponding average.

**Listing 7.** Example of the application of a downsampling method.

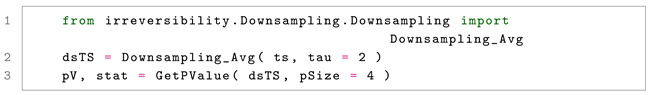



The resulting *p*-value, ≈0.019, is much higher than the original and the test is not statistically significant; this is to be expected, as the logistic map has no multi-scale structure, and its chaoticity implies that memory is lost after a few steps [18].

### 3.4. Manipulating the Irreversibility

As a final point, we are illustrating the possibility offered by the *irreversibility* package in terms of manipulating a time series in order to increase (or decrease) its irreversibility. The idea is to transform the original time series, in this case by applying Continuous Ordinal Patterns [22], to decrease (or, respectively, to increase) the *p*-value yielded by a test, while maintaining a high correlation between the original and transformed time series [48].

The code in Listing 8 starts by creating a random time series, which is by definition time-reversible. The manipulation is then performed by defining the direction of the manipulation (in this case increase = True, hence the irreversibility is increased), the number of independent patterns that are tested (numIterations = 1000), the size of each pattern (pSize = 4), and the maximum *p*-value to be accepted (pvThreshold = 0.01). The results include the manipulated time series, the corresponding *p*-value, and the correlation coefficient between the original and manipulated data. Note that the result, with a *p*-value of ≈0.0013, is highly time irreversible, while maintaining a high correlation with the original (ρ≈0.719).

**Listing 8.** Manipulating the irreversibility of a random time series using COPs.

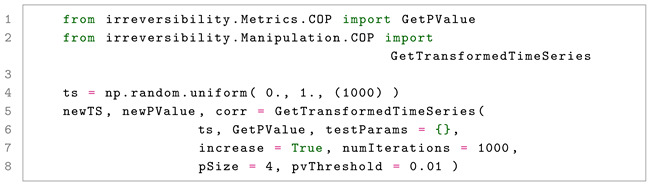



## 4. Computational Cost and Optimisations

**Figure 2:**
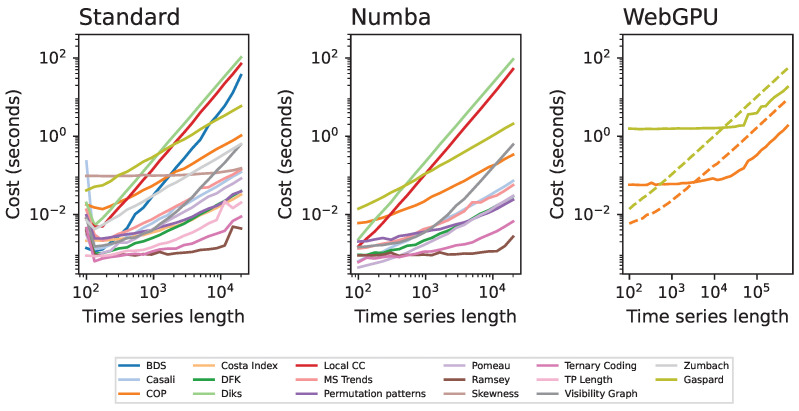
Evolution of the computational cost of all tests as a function of the length of the analysed time series. From left to right, the three panels correspond to results of the standard (i.e., native Python) implementations; the use of the Numba JIT compiled version; and the WebGPU implementation. In the latter case, dashed and solid lines, respectively, correspond to the WebGPU and the Numba version for comparison. Results correspond to the average of 20 independent realisations, on time series generated with a logistic map, on a single core of a 3.8 GHz 8-cores Intel Core i7 CPU.

An important aspect to be taken into account in the analysis of real-world data is the associated computational cost. As can be seen in the left panel of Figure 2, this is highly heterogeneous across metrics, both for any given time series length (with differences of more of two orders of magnitude), and also in terms of the scaling with such length. The use of Numba to precompile the code results in a generalised reduction in the cost, in many cases of one order of magnitude; while not a requisite, the use of this library is highly recommended.

Finally, the right panel of Figure 2 reports the evolution of the computational cost when using WebGPU, for the two tests that currently support it. GPUs present the major advantage of allowing the execution of highly parallelised computations; at the same time, they also introduce an important overhead, as the program has initially to be compiled and initialised. As a consequence, the WebGPU implementations (solid lines) outperform the Numba ones only for very long time series (more than $10^{4}$ points). Furthermore, these results are highly dependent on the available hardware. In short, while the use of Numba is always beneficial, the user has to evaluate the actual added value of WebGPU in each individual analysis.

## 5. Discussion and Conclusions

We have presented *irreversibility*, an open-source Python package for the analysis and manipulation of the irreversibility of time series. At its core is a large set of measures and tests, which have been optimised using techniques like JIT compilation and GPU execution, and which can seamlessly be swapped thanks to their common interface. This allows us to mitigate both the problem of reproducibility, as in many cases software implementations of these tests are not publicly available, and also of their complementarity: the user can easily compare the results of multiple tests, thus obtaining a clearer characterisation of the system under study. The package further supports this endeavour by providing multiple functions to tackle common tasks, e.g., downsampling the time series using different approaches; optimising the parameters of the tests; or manipulating the irreversibility towards a given direction. In short, *irreversibility* is designed to be the foundational toolbox for any researcher interested in the analysis of the time irreversibility of time series.

Besides supporting real-world data analyses, we hope that this package will further help in answering some fundamental open issues on this topic. To illustrate, the large variety of existing tests raises the question of whether some commonalities can be found between them, to possibly construct a taxonomy or even a unified model. Besides time asymmetry, the scientific community has also recently explored the issue of amplitude irreversibility [52,53], i.e., the invariance under a x→−x transformation; the implementations here provided can thus serve as a starting point for evaluating other types of symmetries and their breaking. While these questions have a clear theoretical component, *irreversibility* may be useful to validate their answers.

As a final point, we want to highlight that most implementations of the tests included in *irreversibility* have been built in-house; in spite of our best efforts, errors and bugs may appear. We invite the users to submit bug reports, but also suggestions and modifications, through the corresponding sections of the Gitlab repository hosting the project—see the package documentation.

## Figures and Tables

**Table 1:** Classification of irreversibility tests based on the type of analyses they perform. See main text for details.

Tests	Type of Analysis
COP, Permutation Patterns, Costa Index	Ranking between successive values
Local CC, Visibility Graph	Network embedding
BDS, Casali, Diks	Correlations between data points
DFK, Ternary Coding	Symbolisation of individual values
MSTrends, TPLength	Linear and non-linear trends
Pomeau	Asymmetric function
Ramsey, Skewness, Zumbach	Statistics on data

**Table 2:** List of irreversibility tests included in the package. The right-most columns indicate the type of optimisation available for each test, and the options for calculating the statistical significance—see main text for details. Available options are marked with a X.

Name	Ref.	Optimisations		Stat. Significance		
		Numba	WebGPU	Proportion	Z-Score	Native
BDS	[31]	-	-	-	-	X
Casali	[32]	X	-	X	X	-
COP	[22]	X	X	-	-	X
Costa Index	[33]	-	-	X	X	-
DFK	[34]	X	-	-	-	X
Diks	[35]	X	-	-	-	X
Gaspard	[47]	X	X	X	X	-
Local CC	[24]	X	-	-	-	X
MSTrends	[38]	X	-	-	-	X
Permutation Patterns	[21]	X	-	-	-	X
Pomeau	[19]	X	-	X	X	-
Ramsey	[43]	X	-	-	-	X
Skewness	[44]	-	-	-	-	X
Ternary Coding	[20]	X	-	-	-	X
TPLength	[45]	-	-	-	-	X
Visibility Graph	[23]	X	-	-	-	X
Zumbach	[46]	-	-	-	-	X

## Data Availability

The original contributions presented in this study are included in the article. Further inquiries can be directed to the corresponding author.
